# Neutral-Axis Ti_3_C_2_T_x_/GO Sandwich Sensor with Bending Immunity and Deep Learning Tactile Recognition

**DOI:** 10.3390/s26082471

**Published:** 2026-04-17

**Authors:** Jiahao Qi, Tianshun Gong, Debo Wang

**Affiliations:** 1 College of Electronic and Optical Engineering & College of Flexible Electronics (Future Technology), Nanjing University of Posts and Telecommunications, Nanjing 210023, China; b23020710@njupt.edu.cn (J.Q.); b23020625@njupt.edu.cn (T.G.); 2 College of Integrated Circuit Science and Engineering, Nanjing University of Posts and Telecommunications, Nanjing 210023, China

**Keywords:** flexible piezoresistive sensors, Ti_3_C_2_T_x_/GO, neutral-axis design, bending-immune sensing, 1D-CNN

## Abstract

Flexible piezoresistive sensors are often vulnerable to modal ambiguity and bending-induced drift, both of which can obscure true pressure and strain signals under practical operation. Here, we address these limitations by suppressing bending sensitivity at the device level and disambiguating tactile modes at the algorithmic level. We propose and fabricate a Ti_3_C_2_T_x_/graphene oxide (GO) sandwich sensor in which the conductive network is positioned near the neutral axis, thereby ensuring that bending induces negligible axial strain in the active layer. In contrast, out-of-plane pressing enlarges microcontacts, while in-plane stretching disrupts percolation pathways. We develop a composite-beam model to quantify neutral-axis alignment and the resultant bending immunity, realize the device via a straightforward casting process, and systematically characterize its electromechanical response under bending, pressing, nail pressing, and stretching. To further reduce modal ambiguity and improve tactile recognition, a lightweight one-dimensional convolutional neural network (1D-CNN) was introduced to classify temporal resistance signals from the sensor. Experimental results showed that the 1D-CNN achieved a high classification accuracy of 98.52% under flat-state training and testing conditions, and maintained 96.67% accuracy when evaluated on bending-state samples, demonstrating strong robustness against bending-induced interference. Together, the neutral-axis device architecture and the learning-based inference pipeline deliver high sensitivity to pressing and stretching while markedly suppressing the response to bending, thereby enabling wrist-worn pulse monitoring, soft-robotic joint sensing, and plantar pressure insoles.

## 1. Introduction

Flexible pressure and tactile sensors have attracted increasing attention for wearable health monitoring, soft robotics, and human–machine interfaces because they can conform to curved surfaces and convert external mechanical stimuli into electrical signals in real time [1,2,3,4]. According to their transduction mechanisms, flexible pressure sensors can be classified as piezoresistive, capacitive, piezoelectric, and triboelectric devices, while electrochemical and ion-gradient sensing strategies have also been reported [5,6,7]. Among these approaches, piezoresistive sensors are particularly attractive for intelligent tactile sensing because they provide continuous resistance outputs with simple readout circuits, which are well suited for temporal signal acquisition and data-driven analysis. However, flexible sensors are rarely used under perfectly flat conditions. Incidental bending caused by body motion, surface curvature, or conformal attachment can induce parasitic responses and baseline drift, thereby interfering with signals generated by pressing or stretching [8,9]. This problem is especially critical in piezoresistive sensors because bending directly changes resistance pathways, which can lead to modal ambiguity and reduced recognition reliability.

Various structural strategies, including anisotropic microstructures, aligned conductive fillers, porous architectures, and multilayer designs, have been developed to reduce mechanical cross-talk in flexible sensors [10,11,12]. Nevertheless, bending-induced interference remains difficult to eliminate under complex deformation conditions. In multilayer devices, the position of the active layer is crucial. According to neutral-axis mechanics, when the conductive layer is placed near the neutral plane of the structure, the axial strain induced by bending can be greatly reduced [13,14]. As a result, bending-induced electrical drift can be suppressed at the device level while preserving the responses to out-of-plane pressing and in-plane stretching. Therefore, neutral-axis engineering provides an effective route for mechanically decoupling bending from target tactile signals.

Ti_3_C_2_T_x_ is a promising active material for flexible piezoresistive sensing because of its high electrical conductivity, hydrophilic surface chemistry, and solution processability [15,16]. When combined with GO, Ti_3_C_2_T_x_/GO forms a stable layered conductive network with tunable interflake contacts and interlayer spacing. Its piezoresistive response mainly arises from deformation-induced changes in conductive contacts, tunneling distance, and percolation pathways. Pressing tends to increase microcontact area and decrease interfacial gaps, whereas stretching disrupts conductive pathways and enlarges tunneling gaps. In contrast, when the conductive layer is located near the neutral axis, bending introduces minimal axial deformation in the conductive network and thus causes only negligible resistance variation. Meanwhile, the outer Ecoflex layers provide mechanical support, encapsulation, and conformability, while positioning the conductive layer near the neutral axis [17].

In parallel with advances in materials and structures, machine learning has increasingly been introduced into flexible sensing systems to improve signal interpretation and tactile recognition. For example, Kim et al. developed a hybrid tactile sensor and combined it with deep learning for object recognition, showing that flexible tactile signals can be effectively decoded for intelligent perception [18]. Dong et al. developed a machine-learning-assisted flexible dual-modal sensor for robotic electronic skin, enabling material and hardness recognition during grasping [19]. Tang et al. integrated a textile strain sensor with an AI-based decoding framework for wearable silent-speech recognition, further demonstrating the value of machine learning in extracting discriminative information from flexible-sensor signals [20]. However, most previous studies have mainly emphasized either structural strategies to suppress bending-induced interference or algorithmic methods to improve signal classification. Their integration in a single flexible piezoresistive platform for reliable tactile recognition under dynamic bending conditions remains limited.

**Figure 1:**
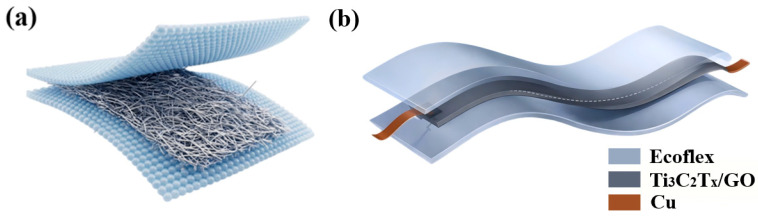
Schematic illustration of the flexible device: (**a**) sandwich structure with a nanofiber network; (**b**) structure of the flexible sensor.

In this work, we propose a neutral-axis Ti_3_C_2_T_x_/GO sandwich piezoresistive sensor for bending-insensitive tactile recognition. As schematically illustrated in Figure 1, the Ti_3_C_2_T_x_/GO conductive layer is embedded between two Ecoflex substrates and positioned near the neutral axis of the composite structure, thereby minimizing bending-induced axial strain and suppressing resistance drift under curvature. As a result, the device exhibits a strongly reduced response to bending while maintaining distinct responses to pressing and stretching. To further reduce modal ambiguity, a lightweight 1D-CNN [21] is employed to classify temporal resistance signals. Different from previous studies that emphasize either structural design or signal recognition alone, this work combines neutral-axis mechanical decoupling and deep learning-based temporal classification in one platform, providing a practical strategy for wearable sensing, soft robotics, and human–machine interfaces under dynamic bending conditions.

## 2. Materials and Methods

### 2.1. Materials

Monolayer Ti_3_C_2_T_x_ dispersion (5 mg/mL, Xinxi Technology, Foshan, China), GO aqueous solution (2 mg/mL, Tanfeng Technology, Suzhou, China), Ecoflex 00-30 silicone elastomer (Smooth-On, Macungie, PA, USA; Part A:Part B = 1:1, volume ratio), Polyethylene terephthalate glycol (PETG) 3D printing filament (melting range: 190–220 °C), silver paste, copper strips.

### 2.2. Sensor Fabrication

The sensor developed in this work features a sandwich structure, consisting of upper and lower flexible substrates, an intermediate conductive layer, conductive silver paste, and copper strips. The flexible substrates can effectively sense external force variations (including pressure, tension, stress, etc.) while protecting the intermediate conductive layer. The intermediate conductive layer is made of Ti_3_C_2_T_x_/GO, which converts external force changes into resistance variations. The Ecoflex layers are not only used for encapsulation and protection, but also help form the symmetric sandwich structure required for the neutral-axis design. In addition, they improve the flexibility and mechanical stability of the device during practical operation.

The sensor was fabricated via a simple casting method using three PETG molds: a lower substrate mold (internal dimensions: 15 mm × 50 mm × 0.2 mm), an intermediate conductive layer mold (internal dimensions: 12 mm × 50 mm × 0.05 mm), and an upper substrate mold (internal dimensions: 15 mm × 50 mm × 0.2 mm).

**Figure 2:**
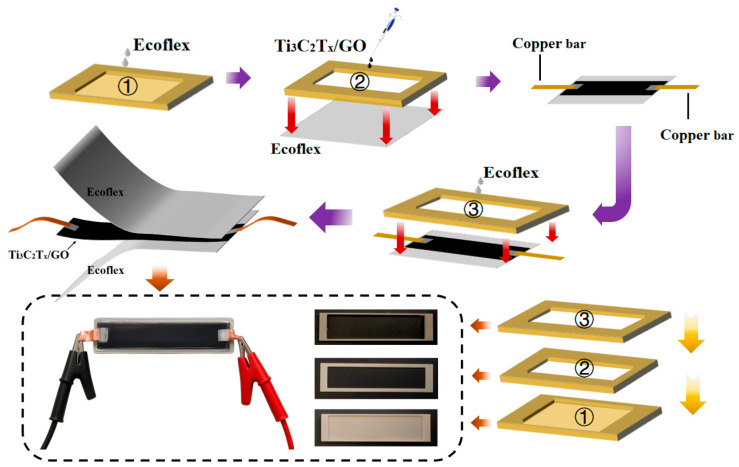
Flowchart for sensor production process and diagram of the physical sensor.

The fabrication process of the sensor is illustrated in Figure 2, as follows:

First, to account for potential losses during handling, 0.2 mL of uniformly mixed commercial Ecoflex (slightly more than the internal volume of the substrate mold) was prepared and carefully poured into the lower substrate mold, ensuring uniform coverage across the mold cavity. Subsequently, the mold was placed in an oven at 80 °C for drying until the Ecoflex was fully cured, forming the lower flexible substrate.

Next, the two prepared solutions were mixed, with Ti_3_C_2_T_x_ accounting for 86 wt% of the total solid content. This composition was selected to balance the electrical conductivity, film-forming uniformity, and structural stability of the conductive layer, where Ti_3_C_2_T_x_ provides the main conductive pathways and GO helps improve the dispersion stability and integrity of the composite film. The mixture was stirred with a glass rod and then subjected to ultrasonic treatment for 15 min to ensure thorough and uniform mixing. The conductive layer mold was placed onto the cured lower flexible substrate, and 0.06 mL of the pre-prepared mixed solution was dropped into the mold. The assembly was then transferred to an oven at 60 °C for secondary drying until the intermediate layer was completely dried. After removing the conductive layer mold, conductive silver paste was applied to both sides of the cured Ti_3_C_2_T_x_/GO film, and copper strips were attached to the silver-paste electrodes as lead wires.

Finally, 0.2 mL of Ecoflex was poured over the intermediate conductive layer to form the upper substrate, and the entire assembly was returned to the oven at 80 °C for drying. Once the Ecoflex was fully cured, the upper substrate mold was removed, yielding the final sensor. Using this casting-based fabrication process, 20 sensors were fabricated under the same conditions, among which 18 devices were successfully obtained with stable electrode connection and intact sandwich structure, corresponding to a fabrication yield of 90%.

### 2.3. Composite-Beam Model

The total electrical resistance R of the conductive Ti_3_C_2_T_x_ network can be described by [22]:

(1) $$R=\rho \frac{L}{A}$$

where ρ is the intrinsic resistivity of the Ti_3_C_2_T_x_ film, L is the effective conductive path length, and A is the cross-sectional area. When the sensor is subjected to mechanical deformation, both L and A vary as a function of strain, while ρ may also change due to the evolution of conductive junctions and tunneling gaps between Ti_3_C_2_T_x_ flakes.

Under tensile or compressive loading, the relative change in resistance can be expressed as [23]:

(2) $$\frac{\Delta R}{R_{0}}=\left(1+2\upsilon \right)\varepsilon +\frac{\Delta \rho }{\rho_{0}}$$

where ε is the axial strain and ν is Poisson’s ratio. The first term accounts for the geometrical effect arising from length and area variation, while the second term represents the intrinsic piezoresistive contribution associated with the contact resistance between Ti_3_C_2_T_x_ nanosheets.

In the case of vertical compression (pressing), interfacial distances between neighboring Ti_3_C_2_T_x_ flakes decrease, resulting in the formation of additional conductive pathways and a reduction in overall resistance. In contrast, during in-plane stretching, the conductive pathways are disrupted and tunneling gaps widen, leading to an increase in resistance. Therefore, $\Delta R/R_{0}$ exhibits opposite trends under compression and tension, forming the basis of the sensor’s piezoresistive response.

**Figure 3:**
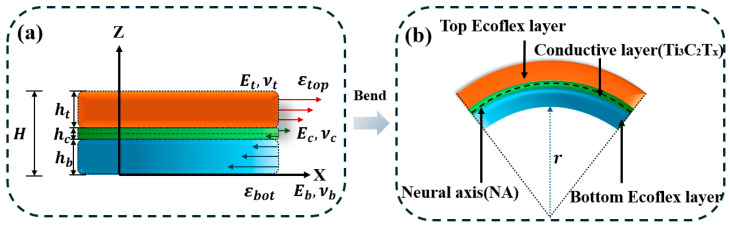
(**a**) Layered sandwich and strain distribution; (**b**) Bent configuration of multilayer structure.

In contrast to uniaxial loading, bending induces a linear strain field across the thickness of the multilayer structure. To quantify the strain within the Ti_3_C_2_T_x_ layer under this bending-induced strain distribution, the neutral-axis theory is employed, as illustrated in Figure 3.

To analyze the mechanical behavior of the proposed structure under bending, we consider a symmetric configuration where the bottom and top layers are composed of Ecoflex (thickness $h_{t}=h_{b}=h$, Young’s modulus $E_{t}=E_{b}=E$, Poisson’s ratio $\nu_{t}=\nu_{b}=\nu$), while the central layer consists of Ti_3_C_2_T_x_ (thickness $h_{c}$, Young’s modulus $E_{c}$, Poisson’s ratio $\upsilon_{c}$). The total thickness of the composite structure is $H=2h+h_{c}$.

Under pure bending, the axial strain varies linearly along the thickness direction and can be expressed as [24]:

(3) $$\varepsilon \left(y\right)=\frac{y-y_{n}}{r}$$

where y denotes the vertical coordinate measured from the bottom surface, $y_{n}$ is the position of the neutral axis, and r is the radius of curvature. The position of the neutral axis is obtained by enforcing the force equilibrium across the composite cross-section [25]:

(4) $$y_{n}=\frac{\sum \bar{E_{i}}A_{i}y_{i}}{\sum \bar{E_{i}}A_{i}}$$

where the effective modulus under plane stress conditions is defined as

(5) $$\bar{E_{i}}=\frac{E_{i}}{1-\nu_{i}^{2}}$$

in which $A_{i}=h_{i}$ represents the cross-sectional area per unit width, and $y_{i}$ is the centroidal coordinate of each layer.

For a symmetric three-layer configuration, the centroids of the constituent layers are defined as follows: the bottom Ecoflex layer at $y_{b}=h/2$, the Ti_3_C_2_T_x_ interlayer at $y_{c}=h+h_{c}/2$, and the top Ecoflex layer at $y_{t}=3h_{c}/2+h_{c}$, where *h* and $h_{c}$ denote the thicknesses of the Ecoflex and Ti_3_C_2_T_x_ layers, respectively. Substituting these parameters into the general expression for the neutral-axis position yields [26]:

(6) $$\bar{E_{i}}=\frac{E_{i}}{1-\nu_{i}^{2}}$$

where $\bar{E}=E/(1-\nu^{2})$ and $\bar{E_{c}}=E_{c}/(1-\nu_{c}^{2})$ represent the equivalent plane-stress moduli of Ecoflex and Ti_3_C_2_T_x_/GO, respectively.

Considering the substantial stiffness mismatch between the soft elastomer (Ecoflex, on the order of MPa) and the rigid Ti_3_C_2_T_x_ (on the order of GPa), i.e., $\bar{E_{c}}\gg \bar{E}$, and the fact that the Ti_3_C_2_T_x_ layer is much thinner than the Ecoflex layers ($h_{c}\ll h$), the terms containing $\bar{E_{c}}h_{c}$ dominate both the numerator and the denominator. Accordingly, equation. (4) can be approximated as [27]:

(7) $$y_{n}\approx \frac{\bar{E_{c}}h_{c}(h+\frac{h_{c}}{2})}{E_{c}h_{c}}=h+\frac{h_{c}}{2}$$

This result indicates that the neutral axis passes precisely through the center of the Ti_3_C_2_T_x_ layer. Since the neutral axis coincides with the midline of the Ti_3_C_2_T_x_ layer, the bending-induced strain in the Ti_3_C_2_T_x_ is given by [28]:

(8) $$\varepsilon_{c}=\frac{y_{c}-y_{n}}{r}$$

where *r* is the bending radius. Consequently, the Ti_3_C_2_T_x_ layer experiences negligible axial strain during bending, which theoretically leads to minimal change in its electrical resistance. This prediction aligns well with our experimental observations and substantiates the effectiveness of the neutral-axis design in decoupling bending deformations from the electrical response of the conductive layer.

### 2.4. Characterization and Measuring Platform

To analyze the microstructure and properties of the material, the samples were characterized by scanning electron microscopy, X-ray diffraction, and Raman spectroscopy.

**Figure 4:**
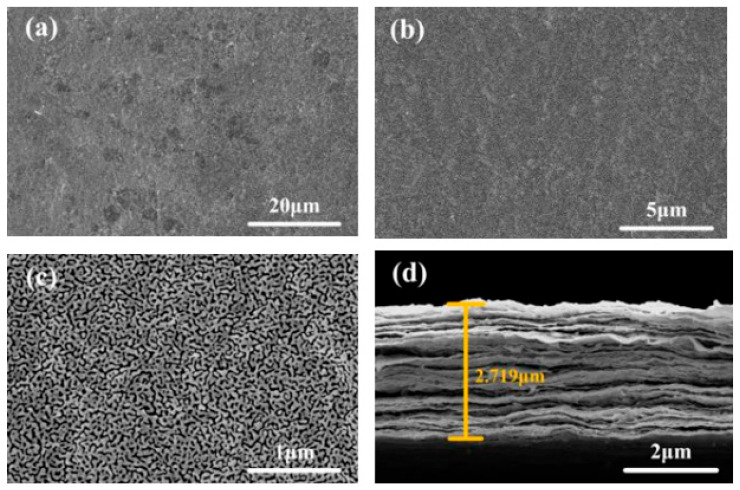
SEM image of the sensor. (**a**) Surface 20 μm magnification; (**b**) Surface 5 μm magnification; (**c**) Surface 1 μm magnification; (**d**) Cross-Section 2 μm magnification.

Scanning electron microscopy (SEM, Nova NanoSEM 450, Thermo Fisher Scientific, Hillsboro, OR, USA) is a specialized observation device that employs a narrowly focused high-energy electron beam to scan a sample. By means of the interaction between the electron beam and the tested material, a series of physical information is excited, which is then collected, amplified, and imaged to characterize the microscopic morphology of the material [29]. Figure 4 shows SEM images of the sandwich sensor at different magnifications. As the magnification increases, the microstructure of the composite film becomes clearer, and the wrinkles in the SEM images become more distinct. Figure 4c shows some clearly visible granular solids, indicating that the Ti_3_C_2_T_x_ material has been successfully doped into the graphene structure, forming a uniform composite structure. As shown in Figure 4d, the cross-section of the sample exhibits a clear two-dimensional layered stacking structure with a film thickness of 2.719 μm, and multi-region measurements indicate the thickness is uniform (2.6–2.9 μm). This two-dimensional layered structure is a typical characteristic of graphene and Ti_3_C_2_T_x_ materials.

**Figure 5:**
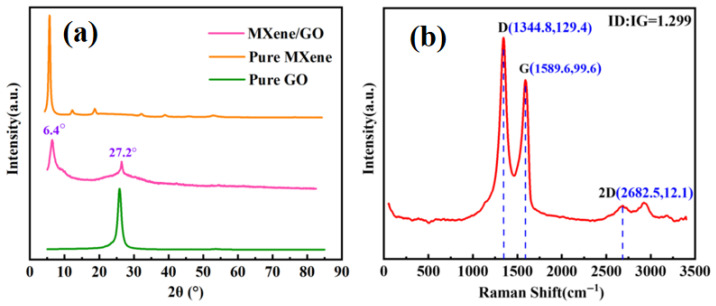
(**a**) XRD pattern of Ti_3_C_2_T_x_/GO structure; (**b**) Raman spectrum of Ti_3_C_2_T_x_/GO structure.

X-ray diffraction (XRD) is a crucial and widely used material characterization technique. Since the wavelength of X-rays is comparable to the size of atoms, the intensity or diffraction pattern of XRD is used to obtain information about atomic structure [30]. Figure 5a shows the XRD pattern of the Ti_3_C_2_T_x_/GO composite structure, where two prominent diffraction peaks can be observed at 2θ = 6.4° and 2θ = 27.2°. Using Jade 6.5 software to analyze the diffraction peaks, it can be concluded that the characteristic peak at 2θ = 6.4° corresponds to Ti_3_C_2_T_x_ material, and the characteristic peak at 2θ = 27.2° corresponds to GO material. The peak width corresponds to grain size and crystallinity. The two prominent diffraction peaks in the figure are narrow and high, indicating that the crystallinity of Ti_3_C_2_T_x_ and GO is quite ideal [31]. Furthermore, no other phases were identified, confirming the purity of the Ti_3_C_2_T_x_/GO composite material and the effectiveness of the synthesis method.

Raman spectroscopy is a non-destructive analytical technique that can reveal key information about the chemical structure, phase and morphology, crystallinity, and molecular interactions of a sample [32]. To further characterize the morphology of the Ti_3_C_2_T_x_/GO composite film, the fabricated film was analyzed using Raman spectrum. As shown in Figure 5b, the Ti_3_C_2_T_x_/GO composite film exhibited three characteristic peaks in the Raman spectrum: the D peak at 1344.8 cm^−1^, the G peak at 1589.6 cm^−1^, and the 2D peak at 2682.5 cm^−1^. The D peak is usually associated with defects, disordered structures, and edge effects in graphite materials, while the G peak reflects the integrity of the lattice structure. The ID/IG ratio is used as an important parameter for evaluating the level of graphitization: a higher ratio indicates a higher defect density and a lower degree of graphitization. The ID/IG ratio measured in this work is 1.299, indicating that the composite film has a high defect density and a relatively low degree of graphitization, which may induce lattice distortion and raise the defect level in the Ti_3_C_2_T_x_/GO structure.

The SEM, XRD, and Raman results not only confirm the formation of the Ti_3_C_2_T_x_/GO composite film, but also help explain its piezoresistive behavior. The SEM images show a uniform layered structure, which is favorable for forming conductive pathways based on interflake contacts and tunneling gaps. Under deformation, changes in these conductive junctions dominate the resistance response: pressing increases effective contact and decreases resistance, whereas stretching enlarges interflake gaps and increases resistance. In addition, GO helps improve the structural continuity and stability of the composite film, while the XRD results further support the formation of a well-defined layered structure without obvious impurity phases. The relatively high $I_{D}/I_{G}$ ratio from Raman analysis indicates the presence of defects and disordered domains, which can introduce heterogeneous contact states and tunneling barriers, making the conductive network more sensitive to microstructural rearrangement under deformation.

**Figure 6:**
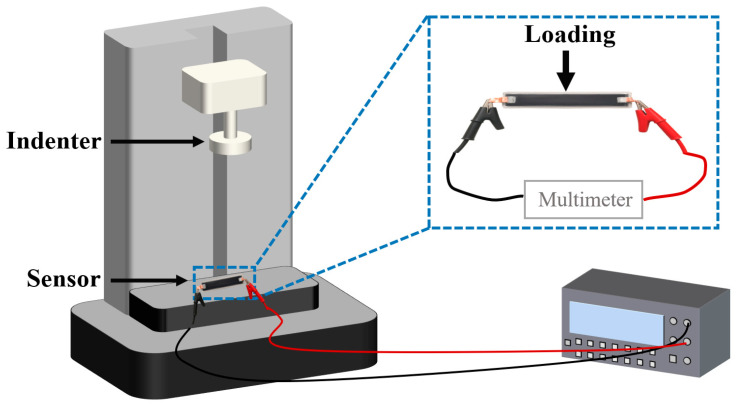
Equipment for sensor measurement.

To guarantee the accuracy and repeatability of the measuring results, a standardized pressure loading system is guaranteed by the electronic universal testing machine, and high-precision digital multimeter (8808A, Fluke Corporation, Everett, WA, USA) is employed to measure the resistance of the pressure sensing units, as depicted in Figure 6.

### 2.5. Deep Learning Pipeline

**Figure 7:**
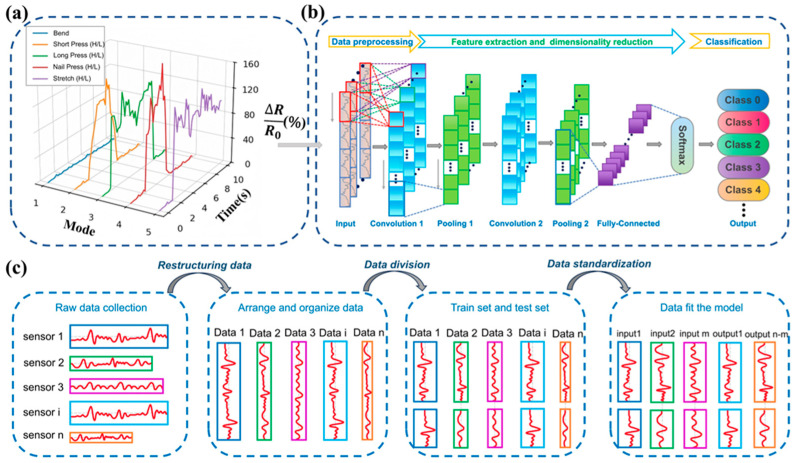
Deep learning pipeline for tactile mode classification using the neutral-axis Ti_3_C_2_T_x_ sandwich sensor: (**a**) Representative resistance–time signals (ΔR/R_0_) measured under different tactile stimuli; (**b**) Architecture of the 1D-CNN used for tactile classification; (**c**) Detailed preprocessing pipeline applied to the raw sensor signals.

To further eliminate modal ambiguity beyond the hardware-level bending immunity, a lightweight one-dimensional convolutional neural network (1D-CNN) pipeline was developed, as illustrated in Figure 7. While the neutral-axis architecture suppresses bending-induced mechanical interference at the device level, algorithmic classification is still required to distinguish complex tactile modes that exhibit similar temporal signal morphologies, particularly between different intensities of pressing and stretching.

Figure 7a presents representative resistance–time responses of the sensor under different tactile stimuli. The signals are expressed as the relative resistance change (ΔR/R_0_), which serves as the input for the machine-learning model. A comprehensive dataset was constructed from nine tactile categories, including bending and eight intensity-differentiated actions: light and heavy short pressing, light and heavy long pressing, light and heavy nail pressing, and light and heavy stretching. For the intensity-differentiated actions, the applied forces were controlled within different ranges. Light pressing corresponded to approximately 1–2 N, while heavy pressing corresponded to approximately 3–5 N. The distinction between short and long pressing was defined by the duration of the applied force. These distinct mechanical interactions generate characteristic temporal patterns in the resistance signals that can be exploited for automatic classification.

The overall deep learning framework is illustrated in Figure 7b. The pipeline consists of three main stages: data preprocessing, feature extraction with dimensionality reduction, and classification. Raw resistance signals are first transformed into fixed-length input sequences before being fed into the convolutional neural network. The CNN automatically extracts hierarchical temporal features from the signals and outputs the probability distribution across the tactile classes through a SoftMax classifier.

The detailed preprocessing procedure [33] is summarized in Figure 7c. Raw resistance signals collected from the sensor are first reorganized and segmented into individual signal samples. Given the data acquisition rate of 100 Hz, a 500-point sliding window is employed to capture the characteristic temporal morphology of each tactile event. A 50% overlap strategy is used during segmentation to enrich the sample representation and improve model robustness. Each tactile class initially contains 70 independent experimental trials, which are expanded into approximately 1890 windowed samples after sliding-window segmentation.

To avoid information leakage caused by overlapping windows, dataset partitioning was first performed at the independent-trial level before sliding-window segmentation. All windows derived from the same raw trial were assigned exclusively to one subset. In addition, five independently fabricated sensors with the same structural design and fabrication process were used in this study. The corresponding device allocation for training, validation, and cross-device external testing is summarized in Table S2 in the Supplementary Information. The test dataset consists of 270 independent non-overlapping samples (30 samples per class) to ensure an unbiased evaluation of the model’s generalization capability. Before training, all signal sequences were normalized using Min–Max scaling to values between 0 and 1, which reduces the influence of amplitude variations and allows the model to focus on temporal morphology.

The architecture of the proposed 1D-CNN classifier is shown in Figure 7b. Each input sample is represented as a 1 × 500 vector. The network begins with a convolutional layer containing 32 filters with a kernel size of 7, enabling the extraction of broad temporal patterns. A max-pooling layer follows to reduce feature dimensionality and suppress noise. A second convolutional layer with 64 filters and a kernel size of 3 further captures higher-level temporal characteristics, followed by a second pooling layer. The extracted feature maps are then flattened and fed into a fully connected layer, where a dropout rate of 0.5 is applied to mitigate overfitting. Finally, a SoftMax activation function outputs the probability distribution across the tactile categories. The 1D-CNN model was trained using the Adam optimizer with a learning rate of 0.001, a batch size of 32, and 500 epochs.

In addition to the proposed 1D-CNN, SVM, Random Forest (RF), and LSTM were implemented as baseline models for comparison. SVM and RF were selected as representative conventional machine-learning methods, whereas LSTM was included as a representative sequence-learning deep model. For fair comparison, all models were evaluated using the same preprocessing pipeline, trial-level data partition, cross-validation protocol, and cross-device test setting. The main hyperparameter settings are summarized in Table S1 in the Supplementary Information. Model performance was evaluated in terms of flat-state accuracy, bending-state accuracy, Macro-F1 score, and 5-fold cross-validation accuracy. These metrics were used to comprehensively assess classification accuracy, class-balanced performance, and model stability.

Categorical cross-entropy was used as the loss function, and early stopping based on validation loss was applied to prevent overfitting. To evaluate the robustness of the proposed sensing–learning system under practical conditions, two comparative testing scenarios were designed. In the baseline test, both the training and test datasets were collected with the sensor maintained in a flat configuration. In the bending-interference test, the model trained on flat-state data was evaluated using a test dataset consisting of 270 independent samples recorded under multiple predefined bending curvatures. Confusion matrices [34] were generated in both scenarios to quantify classification accuracy and potential modal cross-talk. Furthermore, an ablation study [35] was conducted to isolate the contribution of the neutral-axis structure to the classification stability. The recognition performance of the proposed neutral-axis sandwich sensor was compared with that of a non-neutral-axis control device under four different bending curvatures. This comparison demonstrates that device-level suppression of bending-induced signals significantly enhances the robustness of the deep learning classifier in non-planar operating environments.

## 3. Measurements and Results

### 3.1. Sensitivity and Quantitative Evaluation of Bending Suppression

The sensitivity and bending suppression characteristics of the Ti_3_C_2_T_x_/GO neutral-axis sandwich sensor were systematically investigated under different mechanical stimuli. The resistance signals were monitored in real time using a FLUKE 8808A digital multimeter. Repeated measurements showed stable baseline resistance and good device-to-device consistency, indicating satisfactory electrical reproducibility and fabrication reliability.

Sensitivity is one of the key parameters for evaluating the performance of piezoresistive sensors, as it reflects the capability of the device to convert external mechanical stimuli into detectable electrical signals. In this work, the pressure sensitivity S is defined as the relative resistance change per unit pressure and can be expressed as [22]:

(9) $$S=\frac{\partial \left|∆R/R_{0}\right|}{\partial P}$$

where *S* denotes the sensitivity. *R*_0_ denotes the baseline resistance. ∆*R* = *R* − *R*_0_ denotes the resistance variation in the pressure sensor. *P* indicates the pressure applied on the sensor. The formula for the pressure is expressed as:

(10) $$P=\frac{F}{A}=\frac{mg}{A}$$

where *F* represents the force applied to the pressure sensor. *A* denotes the force distribution area. m denotes the total mass of the object above the graphene pressure sensor, and *g* signifies the acceleration.

**Figure 8:**
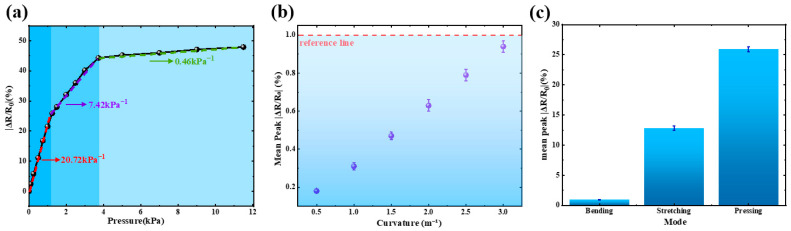
Pressure sensitivity and bending suppression performance of the Ti_3_C_2_T_x_/GO neutral-axis sandwich sensor. (**a**) Relative resistance change versus applied pressure showing three-stage sensitivity behavior. (**b**) Peak relative resistance change under different bending curvatures, indicating negligible bending-induced electrical response. (**c**) Comparison of peak resistance responses under bending, stretching, and pressing stimuli. Error bars represent the standard deviation from repeated measurements.

As shown in Figure 8a, the sensor exhibits an evident three-stage pressure response behavior. In the low-pressure region of 0–1.25 kPa, the relative resistance change increases rapidly, corresponding to a high sensitivity of 20.72 kPa^−1^. In the intermediate pressure range of 1.25–3.73 kPa, the response continues to increase, while the sensitivity decreases to 7.42 kPa^−1^. When the pressure further increases from 3.73 to 11.5 kPa, the resistance response gradually approaches saturation, and the sensitivity decreases to 0.46 kPa^−1^. Such a nonlinear response is typical of piezoresistive sensors based on percolative conductive networks.

The three-stage sensitivity behavior can be attributed to the progressive evolution of the conductive pathways within the Ti_3_C_2_T_x_/GO layer. At low pressures, the interflake distance decreases rapidly under compression, leading to a pronounced reduction in tunneling resistance between adjacent conductive sheets. Since the tunneling resistance is highly sensitive to interflake spacing, even a slight compression can induce a significant resistance change, resulting in high sensitivity in the low-pressure region. As the pressure increases, more physical contacts are established among adjacent flakes, and the conduction mechanism gradually shifts from tunneling-dominated transport to contact-assisted conduction. In the high-pressure regime, the conductive network becomes highly compact, and the contact area approaches saturation. Therefore, additional compression causes only limited changes in the conductive pathways, leading to a much lower sensitivity.

To quantitatively evaluate the bending suppression capability of the sensor, the peak relative resistance changes under different bending curvatures were measured, as shown in Figure 8b. The bending-induced electrical response increases only slightly with increasing curvature and remains below 1% throughout the tested range. This result indicates that bending deformation introduces only negligible electrical interference. Such bending immunity originates from the neutral-axis sandwich design, in which the Ti_3_C_2_T_x_/GO conductive layer is located close to the neutral mechanical axis of the composite structure. As a result, the conductive layer experiences minimal axial strain during bending, thereby effectively suppressing bending-induced resistance fluctuations.

To further compare the responses under different mechanical stimuli, the mean peak relative resistance changes induced by bending, stretching, and pressing were summarized in Figure 8c. The peak response under bending is only about 0.94%, which is significantly smaller than that under stretching (12.8%) and pressing (25.9%). Quantitatively, the bending-induced response is only 7.34% of the stretching response and 3.63% of the pressing response. These results clearly confirm that the proposed neutral-axis sandwich structure can effectively suppress bending interference while maintaining strong electrical responses to functional tactile stimuli.

### 3.2. Measurements of Hysteresis

Hysteresis is another crucial parameter for evaluating sensor performance. In practical operation, the input–output curves measured during loading and unloading are usually not fully coincident, and this difference is defined as hysteresis. The hysteresis effect is mainly associated with the intrinsic properties of the sensitive material and the internal frictional dissipation of the mechanical structure [36]. Excessive hysteresis may introduce response lag and thereby compromise the precision and reliability of the sensing signal. Its mathematical expression can be written as:

(11) $$\gamma_{h}=\frac{∆H_{1}}{Y_{FS}}\times 100\%$$

where $\gamma_{h}$ denotes the hysteresis error. ∆*H*_1_ denotes the maximum hysteresis difference during the measurement period, and *Y_FS_* represents the maximum output value.

**Figure 9:**
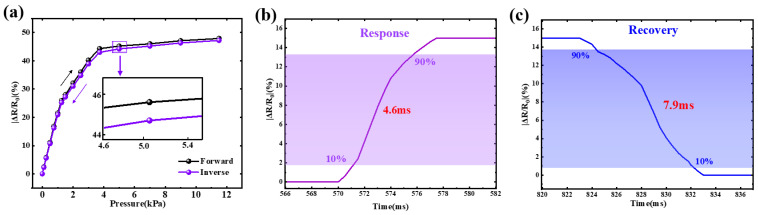
Hysteresis behavior and dynamic response characteristics of the sensor. (**a**) Hysteresis curve obtained from forward and inverse pressure loading cycles. (**b**) Response time measured from 10% to 90% of the peak resistance change. (**c**) Recovery time measured from 90% to 10% of the peak resistance change.

The hysteresis characteristic of the sensor is illustrated in Figure 9a, where the forward and inverse resistance responses under different pressures are compared. It can be observed that the loading and unloading curves are highly consistent over the entire pressure range, indicating good reversibility of the device. A slight deviation between the two curves appears at higher pressures, and the maximum difference occurs at approximately 5 kPa. According to Equation (11), the hysteresis error is calculated to be about 2.1%. The result indicates that the hysteresis error of the sensor is well controlled within 5%, demonstrating excellent repeatability and mechanical stability. This behavior can be attributed to the elastic deformation characteristics of the Ti_3_C_2_T_x_/GO conductive network and the buffering effect of the Ecoflex encapsulation layers, which effectively suppress irreversible structural deformation during cyclic loading and unloading.

The dynamic response characteristic is a key parameter for evaluating the real-time sensing capability of flexible pressure sensors. The response and recovery processes of the fabricated Ti_3_C_2_T_x_/GO neutral-axis sandwich sensor are presented in Figure 9b and Figure 9c, respectively. In practical sensing applications, rapid electrical response to external stimuli is essential for accurately capturing transient mechanical events. Therefore, the response time and recovery time are widely used as important indicators to evaluate the dynamic performance of piezoresistive sensors.

In this study, a controlled pressure loading process was applied to evaluate the transient electrical behavior of the sensor. As illustrated in Figure 9b, the response time is defined as the duration required for the relative resistance change (ΔR/R_0_) to increase from 10% to 90% of its peak value after the application of pressure. The measured response time of the sensor is approximately 4.6 ms, indicating a rapid electrical response to external mechanical stimulation.

**Figure 10:**
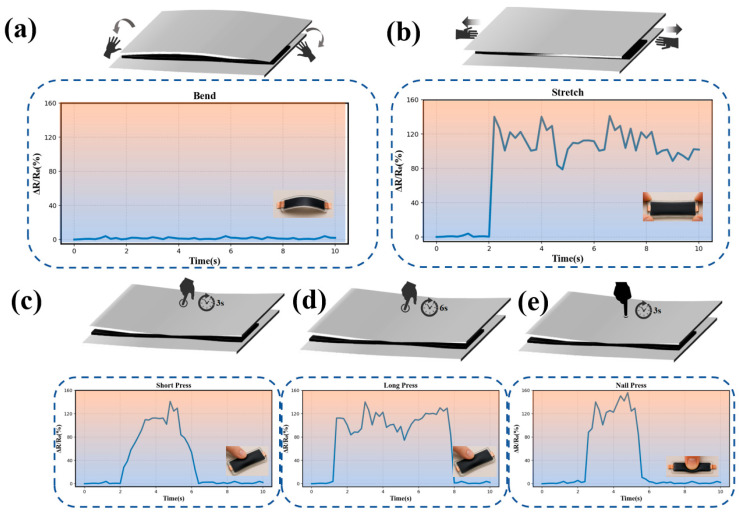
Selective electromechanical response of the neutral-axis sensor to diverse mechanical stimuli. (**a**) Schematic and time-resolved Δ*R*/*R*_0_ curve under pure bending, demonstrating negligible resistance fluctuation (bending immunity). (**b**) Schematic and Δ*R/R*_0_ response under in-plane stretching, showing high tensile strain sensitivity. (**c**–**e**) Schematics and Δ*R/R*_0_ responses under short press, long press, and nail press, respectively, showing distinct, repeatable signals.

Similarly, the recovery process of the device after unloading is shown in Figure 10. The recovery time is defined as the time required for the signal to decrease from 90% to 10% of its maximum value after the pressure is released. The recovery time of the sensor is measured to be about 7.9 ms, demonstrating a fast restoration capability of the conductive network.

The ultrafast dynamic response of the sensor can be attributed to the elastic Ecoflex substrate and the efficient conductive pathways formed by the Ti_3_C_2_T_x_/GO layered network. The high flexibility of the Ecoflex matrix enables rapid mechanical deformation and recovery, while the stacked MXene/GO conductive structure facilitates efficient electron transport during mechanical loading and unloading. As a result, the sensor exhibits excellent dynamic response performance, which is highly desirable for real-time flexible sensing applications.

### 3.3. Selective Electromechanical Response and Bending Immunity

To evaluate the mechanical stability and sensing performance of the as-fabricated neutral-axis sensor, its electromechanical responses toward typical mechanical deformations, including bending, in-plane stretching, and different pressing modes, were systematically characterized, as summarized in Figure 10. Figure 10a displays the schematic illustration and the corresponding time-resolved relative resistance variation (ΔR/R_0_) curve of the sensor under pure bending deformation. Notably, the sensor shows negligible resistance fluctuation during the bending process, manifesting outstanding bending immunity. The negligible response under bending is attributed to the neutral-axis design of the sandwich structure. Since the conductive Ti_3_C_2_T_x_/GO layer is located near the neutral axis, the axial strain applied to this layer during bending is very small, resulting in only a slight resistance variation. Therefore, the device is insensitive to bending but remains responsive to pressing and stretching. This feature is highly desirable for flexible electronic devices, as it ensures stable signal output and avoids signal interference caused by unavoidable bending deformations in practical applications.

In sharp contrast, the sensor exhibits a distinct and sensitive resistance response to in-plane stretching, as demonstrated in Figure 10b. Upon applying tensile strain, the relative resistance changes significantly and synchronously with the applied deformation, indicating high tensile strain sensitivity. This contrasting behavior between bending and stretching confirms the rationality of the neutral-axis structural design, which effectively suppresses the resistance response to bending while maintaining excellent responsiveness to tensile strain.

Furthermore, the sensing characteristics of the sensor under different pressing stimuli were investigated, as shown in Figure 10c–e. The corresponding schematic diagrams and ΔR/R_0_ curves reveal that the sensor generates distinguishable and repeatable resistance signals under short press, long press, and nail press, respectively. Each pressing mode corresponds to a characteristic signal profile with good repeatability and stability, demonstrating the sensor’s ability to identify diverse pressure stimuli. These results collectively verify that the prepared neutral-axis sensor possesses excellent bending immunity and high sensitivity to stretching and pressure, as well as reliable and selective electromechanical response performance under complex mechanical stimuli.

The differences among short press, long press, and nail press can be further understood from the distinct temporal and spatial stress fields imposed on the Ti_3_C_2_T_x_/GO conductive network. Under short pressing, the conductive layer undergoes a transient out-of-plane compression, which briefly increases interflake contact and reduces local tunneling gaps, leading to a rapid resistance decrease followed by prompt recovery after unloading. Under long pressing, a similar compression-induced enhancement of conductive contacts occurs, but the maintained load keeps the conductive network and the surrounding Ecoflex matrix in a deformed state for a longer period, resulting in a more sustained response profile. In contrast, nail pressing applies force over a much smaller contact area and therefore generates stronger local stress concentration and more spatially confined deformation in the conductive layer. This localized compression causes a more pronounced perturbation of interfacial contact states and local conductive pathways in the Ti_3_C_2_T_x_/GO film, thereby producing a sharper and more distinctive temporal signal. These results indicate that the piezoresistive response of the sensor is governed not only by the applied force magnitude, but also by the loading duration and contact geometry, which together determine the evolution of contact resistance and tunneling resistance within the layered conductive network.

### 3.4. Deep Learning Performance

**Figure 11:**
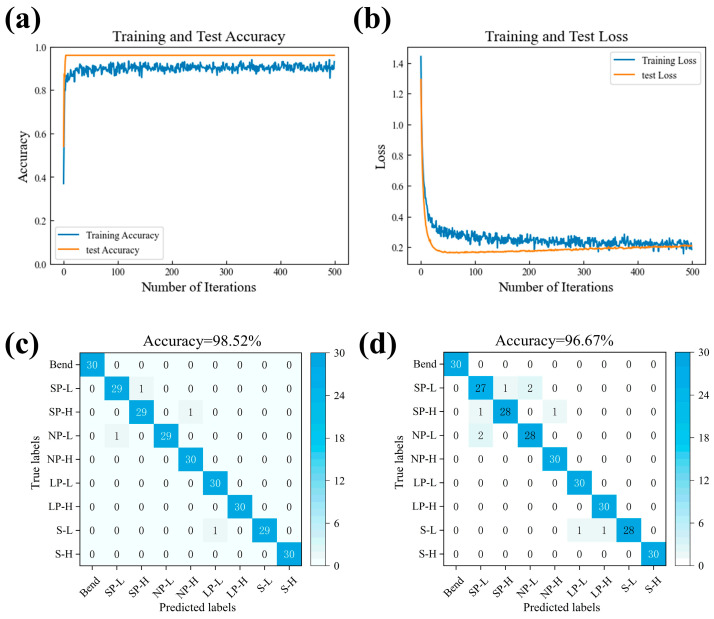
Training performance of the 1D-CNN classifier. (**a**) Training and test accuracy curves under flat–flat conditions; (**b**) Training and test loss curves under flat–flat conditions; (**c**) Confusion matrix for flat–flat training and testing; (**d**) Confusion matrix for flat–flat training and bending-state testing.

The results in Section 3.1 demonstrate that the neutral-axis design effectively suppresses bending-induced signal disturbance while preserving clear resistance responses to stretching and pressing stimuli. Although such device-level selectivity provides an important foundation for tactile sensing, accurate recognition of multiple force modalities still depends on whether their temporal signal characteristics can be robustly separated in feature space. On this basis, we next investigate the deep learning performance of the proposed 1D-CNN framework and assess its classification capability under both flat-state and bending-state testing conditions. The performance of the model was first evaluated under flat–flat conditions, where both the training and test datasets were collected with the sensor in a flat configuration. As shown in Figure 11a,b, the training and test accuracy curves rapidly converge. Correspondingly, the training and test loss steadily decrease and stabilize, indicating that the model successfully learned the temporal patterns of the sensor signals without overfitting. These results reflect the intrinsic learning capability of the network under ideal, interference-free conditions. Classification performance was further examined using confusion matrices, which are presented in Figure 11c,d. In the flat–flat scenario, the overall accuracy reached 98.52%, with minor misclassifications observed mainly between SP-L and NP-L classes. When the model trained on flat-state data was evaluated on bending-state test samples, the overall accuracy slightly decreased to 96.67%, and most misclassifications occurred among SP-L, SP-H, and NP-L categories. This demonstrates that, while bending introduces some interference, the model remains highly robust, benefiting from the sensor’s neutral-axis design.

**Figure 12:**
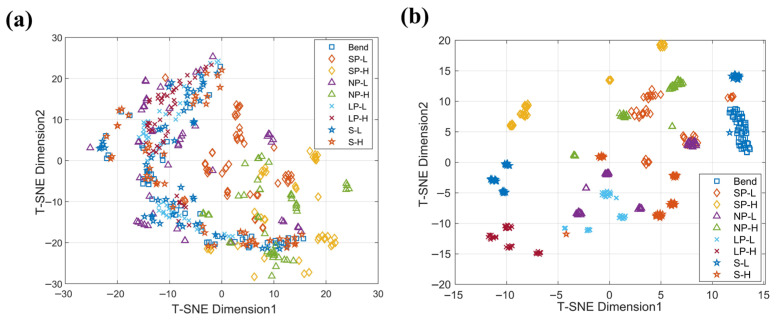
t-SNE visualization of tactile signal features. (**a**) Raw ΔR/R_0_ signals before CNN feature extraction, showing overlapping clusters; (**b**) CNN-extracted features projected via t-SNE, demonstrating improved class separability and clearer cluster boundaries.

The effectiveness of CNN-based feature extraction is evident from the t-SNE visualization of the feature space (Figure 12). Before CNN processing, the 500-point temporal sequences of raw ΔR/R_0_ signals were directly reduced to two dimensions, showing substantial overlap among categories, particularly SP-L, SP-H, and NP-L. After CNN processing, the extracted 64-dimensional features were projected to two dimensions using t-SNE, resulting in well-separated clusters and clearer class boundaries. This indicates that the 1D-CNN successfully captures hierarchical temporal features that distinguish tactile modes. Standard t-SNE parameters were used (perplexity 30, 1000 iterations), providing a consistent and interpretable low-dimensional visualization.

To further validate the effectiveness of the proposed model, comparative experiments were conducted using SVM, Random Forest (RF), and LSTM as baseline methods. As summarized in Table 1 the proposed 1D-CNN achieved the best performance across all evaluation metrics. Specifically, it reached accuracies of 98.52% and 96.67% under the flat and bending states, respectively, which were higher than those of SVM (90.5% and 85.8%), RF (92.8% and 88.1%), and LSTM (95.6% and 91.9%). In addition, the proposed model obtained the highest Macro-F1 score of 0.98, indicating superior class-balanced recognition performance. It is worth noting that all models exhibited lower recognition accuracy in the bending state than in the flat state, indicating that mechanical deformation increased the complexity of signal patterns and made tactile classification more challenging. Nevertheless, the proposed 1D-CNN still maintained the highest bending-state accuracy of 96.67%, demonstrating its stronger robustness to deformation-induced signal variations compared with conventional machine-learning models and the LSTM baseline.

**Figure 13:**
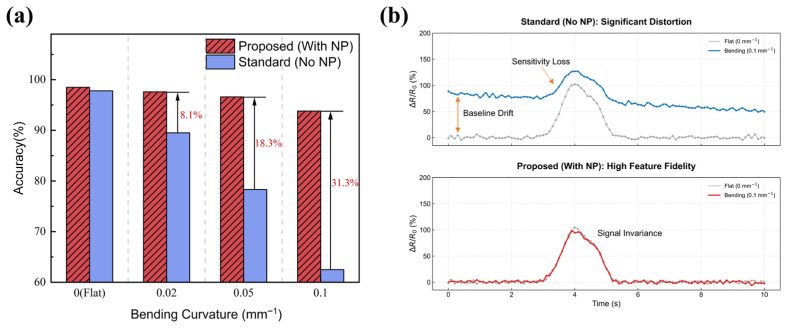
Ablation experiments assessing the effect of the neutral-axis structure on bending robustness. (**a**) Classification accuracy of neutral-axis (Proposed) and non-neutral-axis (Standard) sensors under increasing bending curvatures; (**b**) Signal comparison for Short Press Light: the neutral-axis sensor maintains ΔR/R_0_ stability.

Ablation experiments verify the contribution of the neutral-axis design to classification stability under bending conditions, as depicted in Figure 13. Performance comparison across different bending curvatures shows that the neutral-axis sensor maintains high accuracy (≥95%), whereas the standard non-neutral-axis sensor exhibits substantial degradation, dropping below 85%. Signal traces for short press (light) exemplify this effect: the neutral-axis device shows a maximum ΔR/R_0_ of approximately 1.00 with minimal baseline drift, while the non-neutral-axis device reaches a similar peak (≈1.25) but suffers from more pronounced drift. These observations confirm that the neutral-axis structure effectively preserves signal fidelity under bending, ensuring reliable input for the CNN classifier and consistent tactile recognition. Overall, these results demonstrate that the combination of the neutral-axis Ti_3_C_2_T_x_/GO sandwich sensor and the 1D-CNN classifier achieves accurate, robust tactile recognition, with strong resistance to bending-induced interference and clear feature separability among tactile modes.

## 4. Discussion

Compared with recent MXene-based sensors, multimodal flexible sensors, and ML-integrated tactile sensing systems, the present work is characterized by a balanced co-design of device structure and signal recognition framework [36,37,38,39,40]. As summarized in Table 2, the proposed Ecoflex Ti_3_C_2_T_x_/GO sandwich sensor achieves both structural bending immunity and high ML-assisted recognition accuracy, reaching 98.52% under flat-state testing and 96.67% under bending-state testing. In contrast, some reported MXene-based sensors mainly emphasize high recognition accuracy but lack bending immunity, whereas others achieve deformation tolerance but do not incorporate intelligent classification. Therefore, the main novelty of this work does not lie simply in pursuing the highest single accuracy value, but in establishing a compact tactile sensing platform that can maintain robust recognition performance under mechanically perturbed conditions. This is particularly relevant for wearable scenarios, where incidental bending and deformation are unavoidable and often degrade signal reliability.

The experimental results also provide support for the effectiveness of the neutral-axis design. According to the composite-beam analysis, when the conductive MXene/GO layer is positioned close to the neutral mechanical plane, the axial strain induced by bending should be strongly suppressed. Our observations are consistent with the theoretical prediction and suggest that the conductive layer is effectively decoupled from bending-induced axial strain. Nevertheless, the current mechanical interpretation is still based on a simplified analytical model. More detailed strain-field characterization, direct experimental mapping of the neutral-axis position, and finite-element simulation of the multilayer deformation behavior would further strengthen the mechanical evidence and will be pursued in future work. Relevant prior studies on neutral-plane mechanics in multilayer flexible electronics also support the physical rationality of this design strategy [13,14].

Despite the promising sensing and classification performance, several limitations should be acknowledged. First, although the revised study has strengthened the validation strategy through trial-level data partition, cross-validation, baseline comparison, and multi-device testing, the dataset size and the range of mechanical conditions remain relatively limited. Second, for practical wearable applications, additional challenges such as sweat interference, temperature fluctuation, motion artifacts, and substrate-device coupling on soft skin or textile surfaces may affect signal stability and classification reliability. Third, the long-term reliability of MXene-based devices remains an important concern, since cyclic loading durability, storage stability, humidity exposure, and possible oxidation of MXene were not systematically evaluated in the present study. These issues are crucial for real wearable deployment and should be regarded as important directions for future investigation. Therefore, the present study should be viewed as establishing a deformation-aware sensing and recognition framework, while future work will focus on durability optimization, environmental reliability, FEM-assisted structural design, textile integration, and real-use validation in wearable smart systems.

## 5. Conclusions

This work presents a neutral-axis Ti_3_C_2_T_x_/GO sandwich piezoresistive sensor combined with a 1D-CNN for bending-immune tactile recognition, where locating the conductive layer at the neutral plane achieves negligible resistance drift under bending while maintaining high sensitivity to pressing and stretching—as validated by composite-beam modeling and material characterizations confirming uniform structure and stability. The 1D-CNN classifier realizes 98.52% accuracy in the flat state and 96.67% under bending interference, with ablation tests verifying that this hardware–algorithm co-design effectively preserves signal fidelity and improves classification robustness, thereby solving bending drift and modal ambiguity in flexible sensing for promising applications in wearable electronics, soft robotics, and human–machine interfaces; future work will focus on performance optimization, sensor arrays, wireless integration, and adaptive algorithm upgrades.

## Figures and Tables

**Table 1:** Values for model comparison in tactile recognition.

Model	Flat-State Accuracy (%)	Bending-State Accuracy (%)	Macro-F1	5-Fold CV Accuracy (%)
SVM	90.5	85.8	0.89	89.6 ± 1.8
Random Forest	92.8	88.1	0.91	91.4 ± 1.5
LSTM	95.6	91.9	0.94	94.3 ± 1.2
1D-CNN (proposed)	98.52	96.67	0.98	97.4 ± 0.8

**Table 2:** Comparison of the proposed sensor with representative related works.

Ref	Bending Immunity	Sensitivity (kPa^−1^)	ML-Assisted Recognition Accuracy	Structure Novelty
This work	Yes	20.72(0–1.25 kPa)	98.52% (flat-state test) 96.67% (bending-state test)	sandwich
[36]	No	652.1	ML-enabled real-time blood pressure prediction	textile + serpentine electrode
[37]	No	54.71 (1–10 kPa)	>98%	wrinkle composite (PVA/SWCNT/MXene)
[38]	No	16.7 (<20 kPa)	posture classification demonstrated	paper-based breathable sensor
[39]	No	2.88 (0–300 kPa)	No	layer-by-layer porous TPU/MXene
[40]	No	2.63	No	hollow-substrate MXene/MWCNT network

## Data Availability

Data are contained within the article.

## Supplementary Materials

The following supporting information can be downloaded at: https://www.mdpi.com/article/10.3390/s26082471/s1, Table S1: Main hyperparameter settings of the compared models. Table S2. Summary of dataset partition and device allocation.
