# Supplementary material for: Neutral-Axis Ti3C2Tx/GO Sandwich Sensor with Bending Immunity and Deep Learning Tactile Recognition
Source: Sensors (Basel). 2026 Apr 17;26(8):2471. doi: 10.3390/s26082471 (PMC13120495; doi:10.3390/s26082471)
Supplement: Supplementary file 1 [file sensors-26-02471-s001.zip › sensors-4246904-supplementary.pdf]

Neutral-Axis $Ti_3C_2T_x$/GO Sandwich Sensor with Bending Immunity and Deep-Learning Tactile Recognition

Jiahao Qi[1], Tianshun Gong [1] and Debo Wang [2,*]

[1] College of Electronic and Optical Engineering & College of Flexible Electronics (Future Technology), Nanjing University of Posts and Telecommunications, Nanjing, 210023, China
[2] College of Integrated Circuit Science and Engineering, Nanjing University of Posts and Telecommunications, Nanjing, 210023, China
* Correspondence: wdb@njupt.edu.cn

Table S1. Main hyperparameter settings of the compared models.

Model	Hyperparameter settings
SVM	Kernel = RBF; penalty parameter C = 10; gamma = scale.
Random Forest	Number of trees = 200; maximum depth = 20; minimum samples split = 2; minimum samples leaf = 1.
LSTM	One LSTM layer with 64 hidden units; dropout = 0.5; fully connected output layer; Adam optimizer; learning rate = 0.001; batch size = 32; epochs = 200.
1D-CNN	Two convolutional layers (32 filters, kernel size = 7; 64 filters, kernel size = 3), each followed by max pooling; dropout = 0.5; fully connected output layer; Adam optimizer; learning rate = 0.001; batch size = 32; epochs = 500.

Table S2. Summary of dataset partition and device allocation.

Device	Role	Classes included	Number of trials per class	Used for sliding-window augmentation
Device 1	Training	All 9 classes	70	Yes
Device 2	Training	All 9 classes	70	Yes
Device 3	Training	All 9 classes	70	Yes
Device 4	Validation / CV	All 9 classes	70	Yes
Device 5	External cross-device test	All 9 classes	70	No / only for test segmentation
